# What can we Learn from Patients’ Ethical Thinking about the right ‘not to know’ in Genomics? Lessons from Cancer Genetic Testing for Genetic Counselling

**DOI:** 10.1111/bioe.12272

**Published:** 2016-08-15

**Authors:** Lorraine Cowley

**Keywords:** ethics, genetic counselling, cancer susceptibility, right not to know, genetic test decliners, moral identity, social implications

## Abstract

This article is based on a qualitative empirical project about a distinct kinship group who were among the first identified internationally as having a genetic susceptibility to cancer (Lynch Syndrome). 50 were invited to participate (42 were tested; eight declined genetic testing). 15, who had all accepted testing, were interviewed. They form a unique case study. This study aimed to explore interviewees’ experiences of genetic testing and how these influenced their family relationships. A key finding was that participants framed the decision to be tested as ‘common sense’; the idea of choice around the decision was negated and replaced by a moral imperative to be tested. Those who did not follow ‘common sense’ were judged to be imprudent. Family members who declined testing were discussed negatively by participants. The article addresses what is ethically problematic about how test decliners were discussed and whether these ethical concerns extend to others who are offered genetic testing. Discussions showed that genetic testing was viewed as both an autonomous choice and a responsibility. Yet the apparent conflict between the right to autonomy and the moral imperative of responsibility allowed participants to defend test decliners’ decisions by expressing a preference for or defending choice over responsibility. The ‘right not to know’ seemed an important moral construct to help ethically manage unpopular decisions made by close family who declined testing. In light of this research, the erosion of the ‘right not to know’ in the genomic age could have subtle yet profound consequences for family relationships.

## Introduction

This article explores what can be learned about the ethical thinking of families undergoing genetic testing for a cancer susceptibility syndrome. It is based on empirical data from a qualitative research study^1^ with a particular kinship group, defined genetically as a ‘family’.^2^

Originally, the study aimed to explore, from sociological and anthropological perspectives, the meanings of ‘family’ that participants experienced in the context of their history of genetic investigations. Participants were among the first internationally to have been identified with Lynch Syndrome (LS),^3^ a genetic susceptibility to cancer. They have therefore lived with the implications of this knowledge longer than anyone else, and their experiences form a unique and important case study. A key finding of this research was that participants discussed family members who had declined genetic testing in negative ways, suggesting they felt there was a moral imperative to accept a test. The article addresses what is ethically problematic about the way that test decliners were discussed, and whether these ethical concerns may extend to others who are offered genetic testing. Sharing analyses of participants’ narratives will provide nuanced insights for ethical guidelines that address what is broadly referred to as the ‘psychosocial consequences of genetic testing’. Social sciences have an important role in shaping ethical thinking and the article will make a distinct contribution to this burgeoning body of knowledge.

The article first gives an overview of the study, including the methods used and a brief summary of key findings. This section also addresses what is distinctive about the study family and how they define ‘family’ in this context. The literature on genetic test decliners will be discussed in the section that follows, before analyses of the study's data are given. The analyses draw on several concepts by focusing on how participants discussed family members who declined a genetic test. I shall outline where these data sit within the ethical landscape, and use them to pose questions for current genetic counselling practices. I will conclude with some reflections on the implications of these data for genetic counsellors in their ethical deliberations in the context of genomic medicine.

## About the Study

The study, conducted in the North of England between 2007 and 2012, focused on a family known to a Regional Genetics Service. The term ‘family’ at this stage refers to a genetically defined kinship group as defined by the pedigree that was constructed over decades of genetic research into their history of early onset cancer. This family contributed to research that characterized one of the genes causing LS (hMLh1),^4^ making testing for LS possible and also making them the first to know their genetic risk of LS. They are distinctive when compared to others who undergo genetic testing in contemporary genetic counselling practice.^5^ Their engagement with genetic investigation began in the 1960s when a village GP noticed that multiple family members developed bowel cancer at an unusually young age. He began a genealogical project with some of the family that was continued by genetic services in the 1980s. The genealogy project linked others with cancer to the family.^6^ Some of those who were previously unaware of their biological links became known to each other through this project.

Against this background family members had been offered genetic testing, but not all had accepted. In the study reported here, 50 of those family members were still contactable, and were invited to participate. Of the 50, eight known family members who had declined to be tested also declined to participate in this study. Eight women and seven men, all of whom had accepted a genetic test, agreed to take part; four of the men had the known LS mutation, whilst all others had tested negative. In a series of narrative interviews participants were invited to discuss their experiences of genetic testing and their understandings of family. The interviews were semi‐structured, but interviewees’ own associative trails were followed, allowing them to choose what to tell me. I used photo and graphic elicitation techniques to stimulate responses, for example asking them to talk about family photographs, showing them their genetic family tree, and asking them to construct social maps. Social maps depicted concentric circles within which participants showed who they considered to be closest family (i.e. those placed in the central circle). These narrative methods produced selective accounts, and in their selection of what to tell and with what emphasis, participants created moral identities.^7^

Although the visual methodologies elicited rich narratives about participants’ social practices and meanings of ‘family’, space does not allow further elaboration of these findings here. The focus in this article is on how family members who had declined genetic testing were discussed by participants who had accepted it.

## Test Decliners ‐ what we know from the Literature

There are few studies that focus on those who have declined any genetic test, and this lack is particularly noticeable for cancer genetic testing. An Australian study looked at participants who had declined a test for LS and found that the unanimously reported reason for declining was a potential problem in obtaining medical insurance. While in Australia having a mutation in LS might impede access to health care,^8^ the same is not the case in the UK because of the National Health Service. Interestingly, the focus of critical discourse in the Australian study was insurance companies^9^ because their policies were perceived as preventing individuals from being able to accept a genetic test. An assumption made in that paper is that without insurance barriers, genetic testing for LS would and should be uncritically accepted.

Two ethnographic case studies discussed how genetic test decliners were negatively judged by other members of their families.^10^ In both studies researchers focused on an individual who had declined genetic testing, one for Huntington's disease^11^ and the other for Limb Girdle Muscular Dystrophy,^12^ and found that both individuals were deemed by their families to be avoiding moral responsibility. The studies gave space to test decliners who were previously considered ‘voiceless’^13^ to express their views on living with genetic knowledge. They found that instead of avoiding moral responsibility, test decliners’ decisions about testing were made within a different moral framework and based on alternative rationalities and logic from family members who accepted testing.

Decliners were judged for not engaging with genetic knowledge. However, in fact they had spent time with affected relatives, researched information on the internet or found out about the possibility of genetic testing, so had in fact engaged with genetic knowledge but in a less public way.^14^ Declining a genetic test allowed individuals in both studies to focus their lives on concerns that were generally felt to be legitimate, like caring for their families without the burden of knowledge of an impending debilitating disease. Therefore, their decisions to decline might equally be framed as moral ones. However, in both studies these alternative moral stances were not deemed acceptable by other family members. Test decliners were asked by their families to justify and explain their decisions,^15^ suggesting that there is a dominant moral paradigm within which declining a genetic test does not fit. The authors of this paper make the point that:

Accepting genetics means sliding into a public space where certain moral questions have to be discussed, even if each individual is basically free to make his or her own choice. Genetics, simply because it is public, imposes the category of moral action – the one which implies actors endowed with a capacity for deliberation and decision‐making, who assume full responsibility for their acts.^16^

These cases demonstrate how looking after one's family is morally framed in a genetic context. Both those who accept and those who decline a genetic test could be framed as acting morally, although in the cases illustrated those who declined were not. This suggests that what is being contested here is not *whether* one looks after one's family but *how*. In these case studies, genetic testing is being used as shorthand for doing that. The next section considers how my data contribute to these debates by focusing on the way in which interviewees discussed those who declined genetic testing for LS.

## How LS Interviewees Framed the Notion of Moral Imperatives

It was only when considering interviewees’ narratives about choice in genetic testing that ‘moral imperatives’^17^ about testing became apparent. My interest in morality here is not focused on philosophical understandings of what makes someone or something moral, but rather it draws on sociological and cultural understandings of how moral tales are important to people's sense of identity. In considering participants’ decision‐making in their relational networks I was interested in their ‘ordinary’ ethical approach,^18^ that is, what issues in genetic testing they found important and how they expressed and developed their opinions and arguments.

Some participants’ narratives showed a felt absence of choice despite their own belief in choice overall. In considering genetic testing through a lens of choice, I was struck by the emphasis on the family's role in participants’ constructions of notions of morality. Even when choice was discussed by participants as being important enough to be a fundamental right, my analyses showed that the idea of choice in genetic testing within families was, paradoxically, negated by referring to moral imperatives to have a test. These moral imperatives were expressed in a hierarchical framework. First, all participants discussed a responsibility to be tested on behalf of their children. Second, they all discussed a responsibility for self‐care, articulated through lay health beliefs. Lastly, eight participants discussed a sense of duty to advance medical research. Collectively, the overarching theme of the interviews was the importance of ‘morally’ looking after one's family, however family was defined.

Some participants did not engage with forming an argument about the choice to have a genetic test since it appeared to them to be beyond choice and something that was incomprehensible to decline, a ‘no brainer’. One participant, for example, said it was the ‘obvious thing to do’. Similarly, another did not see choosing to decline a test as a possibility, since those who chose not to be tested were choosing ‘to live in ignorance’ and this was considered unacceptable by him/her.^19^ In short, their views would seem, on first analysis, to question whether declining a genetic test is a moral or ethical position. These views are unpacked further in the following section.

Test decliners were defined by all participants as ‘ostriches’, characterized by the phrase ‘those that bury their heads in the sand’. They were framed as morally weak at best and neglectful at worst, and were perceived as lacking courage (‘They'll not confront anything’); test decliners were ‘frightened of going’ to have a genetic test. The language used to describe those who would not or could not engage with genetic knowledge was telling. Regardless of their own test results, participants described test decliners either explicitly or implicitly as ‘silly’, ‘stupid’, ‘selfish’, lacking in character, fearing the truth, illogical and cowardly. All of these characteristics are uncomplimentary at best and morally damning at worst. Given that the quality of people's lives depends greatly on the quality of their social relations, then to paraphrase Sayer, this must be a thing that matters to people and is therefore worthy of social science's ethical attention.^20^

Participants’ judgement of test decliners was narrated differently depending on the relationship between them. I focus here on examples that illustrate how moral judgement was discursively managed by family members who were troubled by a test decliner's decision. Family members still constructed their family as close whilst managing what was framed as a morally suspect behaviour – declining a test – depending on how close they felt their social relationship to be. This illustrates the efforts participants made to maintain the quality of their relationships despite behaviour of which they disapproved, strongly signalling that maintaining closeness mattered despite the negative moral judgment.

Diane, a participant who did not have the mutation, believed that genetic testing was an individual choice but was nevertheless troubled that her parent and aunt had exercised that individual choice to decline testing. This was problematic for Diane and her siblings because they all wanted to know their genetic status and therefore agreed to be tested. Of her parent's decision to decline she said:

Diane: Well, it was their [parent and aunt's] belief I suppose and their opinions and all that, but I mean, it didn't really help you.

Me: So how did that make you feel about your [parent] and your Auntie?

Diane: I think [sigh] I think initially we were all, probably all [number] of us [Diane and her siblings] were a little bit annoyed. But then I think they were annoyed with us for going to get tested.^21^

Whilst it may have been ‘an individual choice’, this account gives a sense of the tensions that existed between those who chose to be tested and those who chose against. Accepting that choice is rarely completely autonomous but is contingent upon many factors, it is interesting that the individuality of choice was still upheld here, even though contingencies of choice such as a sense of family obligation or sensitivities to others’ needs may have been present but were not perceived as having been acted upon. Diane indicated that family dynamics were disturbed when individuals exercised their choice to be tested or not. For Diane, her parent's decision to decline was framed as being unhelpful to her because she then had to go through the testing process (and was shown not to have the mutation). She felt that her parent's choice should have been morally contingent upon the sense of family obligation in which, in having a test, her parent would be putting the needs of his/her children above his/her own. Whilst from Diane's perspective her parent failed to fulfil this obligation, she later framed her parent's decision as having consequences not only for her, but also, since her parent was deceased, for medical and family research (‘now we'll never know’).

Another interviewee, Fran, who did not have the mutation, discussed how her parent did not want to engage with genetic testing because s/he ‘couldn't cope with it’. Despite this s/he still went ahead with the test but did not want to be told the result. It is interesting that Fran's parent still had the test but managed the situation by requesting that his/her results should not be told to him/her. I shall return to this in a later section. Fran compared herself to her parent:

Fran: I would prefer to know… I suppose [it's] just the way you're born isn't it? Just because I think if you know things you can prepare for them or, do what you want, you know. Where my [parent] would rather not and just carry on.

Fran excused her parent for not wanting to know because it was just the way s/he was born, thereby avoiding the negative associations with others who did not want to know.

Contrast this action with another participant's account of a parent declining a test. Frank illustrated how declining a test was beyond his parent's control because of upbringing:

Frank: So I think [s/he] was brought up with that sort of generation and that mentality you know? And it was sort of like, I don't know, it was almost sort of fate, what will be, will be type of thing. So I can understand from that point of view. I mean we call, or I call that sort of burying your head in the sand but… it's the way… probably the way [s/he'd] been brought up and what [s/he] had seen you know? It was [his/her] way of dealing with it I guess. So I could understand [his/her] point of view.

This quote illustrates an intergenerational difference in approaches to genetic knowledge. In other parts of the interview Frank's parent is discussed as having witnessed the painful death of his/her own parent and the offer of genetic testing was a reminder of this experience: ‘It brought up all the old things [s/he] had. Like [his/her] own [parent] died with it.’

There are multiple accounts of this scenario in the data, older generations having witnessed more cancer deaths with little palliation of symptoms. Thus, previous generations were framed as powerless and therefore non‐culpable in their fate whereas, paradoxically, the present generation does have a ‘choice’ but by making the ‘wrong choice’, i.e. declining intervention whether it is a genetic test or screening interventions such as colonoscopy, become culpable in their fate. By virtue of their experience and the participant's more positive disposition towards this relative, test decliners who were discussed in this way were absolved from moral accountability and became objects of pity and frustration; pity because their trauma was acknowledged and frustration at their lack of action to change their fate. This has resonance with the concepts of brute luck and option luck as discussed in Denier and Hammond's work.^22^ In the context of reproductive technologies, these authors discussed what they call fundamental distinctions between what we are responsible for doing, or deciding, and what is given to us. They considered the distinctions between chance, identified as brute luck (something that we are born with and have no control over), and choice, identified as option luck (where a test or intervention exists that can change or control what we are born with). Ways in which individuals self‐identify as ‘lucky’ or ‘victims of fate’ may recursively influence how they relate to the notion of choice. From my data, test decliners were constructed in a negative light because a genetic test was available. If freedom of choice to decline a genetic test is considered as a right it was paradoxically only portrayed as morally valid when knowledge was unavailable.

While the above accounts rely heavily on the perceived obligation of parents to engage with genetic testing, it is interesting that from the point of view of clinical practice, the concept that there is an imperative to be tested for the benefit of the children is false, since parents could decline a test without obstructing their children's ability to obtain that information for themselves. Additionally, whether a parent knows their genetic status or not does not affect whether their children have inherited the same mutation. If choice in genetic testing is just about logic, Diane should not have been troubled by her parent's choice to decline. If Diane's parent did not have the LS mutation, then her own test was unnecessary but nevertheless Diane was still able to have a test. Participants framed ‘choice’ as an important personal and individual right. Within a liberal rights framework,^23^ a right to choose, as it ostensibly operates in the domain of genetic testing,^24^ means that Diane's parent's decision to decline testing should not be considered problematic. Thus although ‘choice’ seemed important and all participants defended individuals’ right to choose, those choices were only morally valid if the contingencies of familial obligation had been observed. Fran's parent who was tested against her own preferences for herself but not against her will for the benefit of her family, is one example. This leads to questions about the adequacy of the term ‘free from coercion’ within the code of ethics for genetic counselling.^25^ In counselling someone like Fran's mother, the genetic counsellor might be aware of tacit coercion from the family. In negotiating the withholding of results from a hesitant volunteer, the genetic counsellor may be complicit in that coercion.

In participants’ accounts genetic testing was the appropriate moral action to take. Looking at the family history and their accounts of how it was before genetic testing, ‘they were all dying’ (Alan), or ‘the family were going and saying, well, we think we've got cancer you know and doctors were saying to them all, ‘no’ you know. You can't have’ (Fran). Key medical figures, like their GP who began collating their history, were perceived as going beyond the call of duty to find a cause of their relatives’ untimely deaths from cancer. This generated something akin to a sense of obligation to be tested as reciprocation for the time, effort and commitment that had been invested in this family by those key medical figures.

In referring to the rights of autonomy or self‐authorship, participants maintained their moral identities by signalling an apparent acceptance of difference in others but simultaneously negatively judging the actions of those who failed to act in the expected way towards themselves, children, or research. Relatives who declined were seen as neglectful in their actions since they ‘chose’ not to be tested. For interviewees, the only way to construct a moral self‐identity in the context of a genetic test was to be tested; declining a test, on the grounds of avoiding a burden of knowledge, was unacceptable and immoral because it was ‘callous’ and ‘selfish’. Yet positioning the choice not to know as a moral right still had value for interviewees in managing their approach to family members who declined. The right not to know allowed interviewees, who negatively judged family for declining testing, to claim a moral position in doing so.

## Discussion

The study emerged out of an interest in the effect of participants’ involvement in medical research and knowledge production on their senses of individual and kinship identity. The families studied provide a particular context that may well have some bearing on the participants’ views of genetic testing.

The data show that family relationships were put under pressure when some family members declined to accept genetic testing. Interestingly, only two of the eight who declined testing responded to the initial invitation letter and none participated in the study. Thus those who declined to be tested had no representative voice to give a counter‐narrative in the data. Genetic test decliners’ minority voices remained unrepresented in the study. If test decliners did not contribute to the study because they felt negatively judged, then this poses challenges for ethical thinking about those who decline in contemporary approaches to genomic medicine.

Test decliners may be marginalized by the power dynamics and cultural norms of a health system where those offered genetic testing for an adult onset disorder ostensibly should be able to choose freely either to know this information or not. However, although premised on the principle of autonomy, the ‘right not to know’ in genetic testing has been hotly debated^26^ and some have concluded that the ‘right not to know’ does not trump the ‘right to know’, when the right to know is framed as a morally relevant consideration.^27^ These debates have been contextualized and historically located in a field of emerging technological advances; to an extent, technology is eroding the right not to know. For example, in cancer genetics the ability to identify a genetic mutation has often been dependent upon a relative affected by a condition giving a DNA sample to test for an underlying genetic cause.^28^ Those being asked to give a DNA sample to try to identify a familial mutation have been described as holding the ‘trump card’.^29^ The idea of holding a trump card introduces a different power dynamic to family relationships. If the trump card holder does not want to know their genetic status, they may decline to give their DNA. Some consider that in claiming a right not to know, test decliners are potentially harming those who do want to know.^30^ The test decliner's position has been problematized and criticised as being unethical on the grounds of a right to autonomy for all.^31^ It has been suggested^32^ that in such cases, the rights of those seeking genetic information are compromised because information is being withheld such that they cannot act autonomously: since genetic information is a prerequisite for future autonomous decision‐making, by choosing not to be tested test decliners undermine others’ capacity for making autonomous decisions. To defend a choice that compromises autonomy on the grounds of autonomy is referred to as the incoherence objection and is therefore also considered problematic. However, if autonomy is reframed as self‐authorship, in which choices are compatible with an individual's views and beliefs, then the incoherence objection can be refuted.^33^ A test decliner can coherently have self‐authorship regardless of perceived limitations on future decision‐making by themselves or others.

Since the application of whole genome and whole exome sequencing (WGS; WES) the need to test samples (including from those who would rather not know) is less pressing but not redundant. WGS and WES produce copious data, making interpretation problematic. Comparative genome sequences from those affected by the conditions being interrogated are still needed to validate reported results. Therefore, those ‘trump card’ holders who choose not to know may continue to be viewed by critics of the right not to know as having a contestable position. In my study, test decliners did not genuinely hold trump cards since their relatives could still be tested, but declining remained contestable to participants on the grounds that being tested was a familial duty. If this view is widely held in a society where genomics provides more opportunities for genetic testing, then the right to decline is potentially either untenable, or troubling for family relationships.

Genomics knowledge increases the potential to confront those who would rather not know their genetic risk. Genomic technologies produce unlooked for findings, such as cancer susceptibility genes that have actionable clinical relevance to those who were tested and their families. Whether the person who sought a test, or their families, are prepared and want to know these findings is questionable. Negotiating the limits of what *can* be known and what an individual *wants* to know is a precarious ethical situation both for genetics professionals and for those undertaking genetic testing. Non‐disclosure could be problematic for genetics professionals, when working within medical ethical boundaries based on the principles of autonomy, beneficence, non‐maleficence and justice. Erez makes the point that respect for autonomy is ethically important but not absolute and must be balanced against non‐maleficence and professional integrity.^34^

Whilst it may be considered reasonable, and perhaps to some imperative, to give a patient genetic information that they did not want if it saved them from harm, what constitutes harm in this situation would be defined by the medical professional. Cancer susceptibility genes predict probabilities of developing disease, not certainties. As we have seen from my study, for some the potential psychological burden^35^ of this information is a poor trade‐off for the return of an arguably limited ability to prevent and treat early onset disease by means that are available whether or not a genetic test has been used. The test decliner in the age of genomic medicine is therefore in a precarious situation, since in not engaging with genetics services, where their desire not to know may be negotiated, they are at least once removed from the interface of this ethical dilemma. Faced with the full power of advances in genomic medicine, it may be difficult and in some cases impossible to stand against such a force. This may result in the marginalization of test decliners, and if the goal of medicine is to enhance rather than diminish lives, then this is problematic.

## Conclusion

Individuals within families, however they are defined, will express different needs in relation to knowing about inherited genetic mutations. Genetic counselling is predicated on the principle of individual autonomy. This poses inherent tensions in how genetic knowledge comes to be known and shared within what are called ‘families’. This article set out to address what is ethically problematic about how test decliners were discussed in a distinct family with a genetic susceptibility to cancer. Although I was not part of their pre‐genetic history, the way that participants presented their stories may have been influenced by my status as a genetic counsellor turned researcher. Since narrative methods can prioritize moral identity values,^36^ participants might not have framed a moral agenda around declining a genetic test if different methods had been deployed. This article proposed that, within this distinctive study family, it meant more to decline a test because of their pioneering position in the history of LS. If their pioneering identity was the most influential factor in the unquestioning conviction that to have a genetic test was morally the right thing to do then the perceived marginalization of test decliners is unlikely to be common because this pioneering position will not be replicated. Furthermore, the data uncovered a series of (mis)understandings about genetic testing that, if considered using the same logic with which they were expressed, might weaken the moral imperative to be tested. Whether explication of these (mis)understandings might influence how participants viewed having a genetic test, or more pertinently whether it would change their view of those who declined testing and potentially avoid family disharmony, remains moot.

Other studies had highlighted how genetics can be a domain in which one's relationships and human entanglements are publicly dissected, and one's approach to having genetic knowledge cannot be accepted as a ‘free’ choice but requires explanation. The experience of test decliners in Australia suggests that avoiding medical insurance penalties was an easier and socially legitimate explanation that could be safely voiced without fear of negative judgment. Whether test decliners in the LS family were marginalized and felt unable to express what looks like a minority view is not known. Their voices are missing from this article and elsewhere, and this raises concerns for ethical practice in the age of genomics where the boundaries of what is sought and what can be known are blurred. The boundaries are more indistinct for those who choose not to know, and without engagement with genetics professionals, non‐negotiable.

The data showed that genetic testing was viewed as both a choice and a responsibility. Yet the apparent conflict between rights to autonomy and the moral imperatives of responsibility allowed scope to prefer or defend choice over responsibility or responsibility over choice. The right not to know seemed an important moral construct for managing unpopular decisions of test decliners who were also close family members. Eroding the right not to know in the genomic age might therefore have subtle yet profound consequences for family relationships.

Clearly, my participants’ common experiences of genetic investigation over three decades are likely to be different to those who seek genetic knowledge in the early 21^st^ century. However, whilst their processes of knowing may be unique, the strength of the data lies in their stories of troubled or changed family relationships which continue to have resonance for others seeking genetic diagnoses for familial cancer susceptibility. What is important information for families faced with genetic testing goes beyond the timing or process of the test; also important are insights into the long‐term consequences for family relationships.

These insights contribute to important narratives about cultural engagement with genetics and have implications for practice in the genomic era. The data raise questions about the marginalization of test decliners within families, leading to further questions concerning the adequacy and meaning of the term ‘free from coercion’ within the code of ethics for genetic counselling. Since any deployment of genetic knowledge is a moral process, I conclude that honest communication about the limitations of what is offered in genetic and genomic testing is vital. Honest communication about limitations would go some way to address misunderstandings and thereby influence what can be morally claimed in seeking and having genetic information. For example, the limitations to predicting, preventing or ameliorating disease may influence whether having a genetic test is framed as a ‘no brainer’. In this way, those who decline will have more support and space in which to consider their decision and will not risk being judged negatively by family members who want and need to know. In making space for the moral agenda in the clinic, those who would rather decline a genetic test can openly express their morally acceptable alternatives. Whilst there can be varied reasons why the right not to know is a marginalized position, holding that position has an impact on family relationships. In communication about genetic testing what is needed is an approach that is sensitive to the ways in which decisions to test or not to test are both socially located and socially consequential.

## Footnotes

1

L. Cowley , 2012 Genetics and Kinship: Finding Morality at their Intersection In Geography Politics and Sociology. British Library Ethos: Newcastle University: 275.

2
Participants did not always self‐identify with each other as family. Some only became aware they were biologically related through medical investigation of their cancer history. To the medical profession they were defined as a 'family’.

3
Lynch Syndrome (LS) is a dominantly inherited cancer predisposition syndrome thought to account for approximately 5% of all colorectal cancer. Those with LS have up to an 80% lifetime risk of bowel cancer and cancers of the digestive or urinary tract. Additionally, women have an increased risk of gynaecological cancers. H. Lynch, et al. Hereditary Colorectal Cancer: An Updated Review. Part II: The Lynch Syndrome (Hereditary Nonpolyposis Colorectal Cancer). *Gastroenterol Hepatol* 2005; 1: 117–133.

4
R. Kolodner et al. Structure of the Human MLH1 Locus and Analysis of a Large Hereditary Non Polyposis Colorectal Carcinoma Kindred for MLH1 Mutations. *Cancer Research 1995*; 55: 242–248. 7812952

5
Those undertaking genetic testing for LS in current practice would usually see a geneticist or counsellor three times over three to six months. This varies depending on local policies and practices about predictive genetic testing.

6
G. Dunstone & T. Knaggs. Familial Cancer of the Colon and Rectum. *J Med Genet* 1972; 9: 451–456. The family concerned has been discussed in the medical literature. Some family members have contributed to television documentaries. Care has been taken to ensure anonymity and de‐identification of individuals by using pseudonyms and de‐gendering relationships within quotes. Genetics colleagues who have proof‐read quotes were unable to identify participants they would usually know. 10.1136/jmg.9.4.451PMC14690534646555

7
J. Singer. Narrative Identity and Meaning Making Across the Adult Lifespan: An Introduction. *J Pers* 2004; 72: 437–459; Cowley, *op. cit*. note 1. For further consideration of narrative methodologies and moral identities. 10.1111/j.0022-3506.2004.00268.x15102034

8

L. Keogh et al. Is Uptake of Genetic Testing for Colorectal Cancer Influenced by Knowledge of Insurance Implications? MJA 2009; 191: 255–258. 1974004510.5694/j.1326-5377.2009.tb02778.x

9
Ibid.

10
L. Huniche. Moral Landscapes and Everyday Life in Families with Huntington's Disease: Aligning Ethnographic Description and Bioethics. *Soc Sci Med* 2011; 72: 1810–1816, M. Callon & V. Rabeharisoa. Gino'sLlesson on Humanity: Genetics, Mutual Entanglements and the Sociologist's Role. *Econ Soc* 2004; 33: 1–27. 10.1016/j.socscimed.2010.06.03920724050

11
Huniche, *op. cit*. note 11.

12
Callon & Rabeharisoa, *op. cit*. note 11.

13
Huniche, *op.cit*. note 11, p.1812.

14
Ibid: 1812.

15
Callon & Rabeharisoa, *op. cit*. note 11, p.8.

16
Ibid: 19.

17
‘Moral imperative’ denotes how participants strongly emphasized the decision to have a genetic test. Some were passionate about it and all wanted to emphasize the importance (in their view) of accepting a genetic test. Alternative terms such as 'moral reason’ do not capture the passion with which they expressed their views.

18

S. Banks , JL. Scully , T. Shakespeare . Ordinary Ethics: Lay People's Deliberations on Social Sex Selection. New Genet Soc 2010: 136

19
Dunstone & Knaggs, *op. cit*. note 6.

20

A. Sayers . 2011 Why Things Matter to People. Social Science, Values and Ethical Life. Cambridge: Cambridge University Press: 7

21
This suggests that test decliners used a different moral framework and made negative judgments about those who were tested. Although interesting there are no further data with which to explore this suggestion.

22
Y. Denier. From Brute Luck to Option Luck? On Genetics, Justice, and Moral Responsibility in Reproduction. *J Med Philos* 2010; 35: 101–129, J. Hammond. Genetic Engineering to Avoid Genetic Neglect: From Chance to Responsibility. *Bioethics* 2010 24: 160–169. 10.1093/jmp/jhq00720181644

23

T. Beauchamp & J. Childress . 2001 Principles of Biomedical Ethics. New York: Oxford University Press.

24

J. Haydon . Genetics: Uphold the Rights of all Clients to Informed Decision‐making and Voluntary Action. Nurs Stand 2005; 20: 48–51. 1622319510.7748/ns2005.09.20.3.48.c3965

25
AGNC. 2011*. Code of Ethics for Genetic Counsellors* AGNC Website: Association of Genetic Nurses and Counsellors (AGNC). Available at: http://www.agnc.org.uk/about-us/code-of-ethics [Accessed 12 Nov 2015].

26
T. Takala. The Right to Genetic Ignorance Confirmed. *Bioethics* 1999; 13: 288–293, T. Takala & M. Hayry. Genetic Ignorance, Moral Obligations and Social Duties. *J Med Philosy* 2000; 25: 107–113, J. Raikka. Freedom and a Right (Not) to Know. *Bioethics* 1998; 12: 49–63; J. Solbakk, S. Holm, B. Hofmann, eds. 2009. *The Ethics of Research Biobanking*. Dordrecht: Springer.

27
H. Widdows. Conceptualising the Self in the Genetic Era. *Health Care Analysis* 2007; 15: 5–12, J. Harris & K. Keywood. Ignorance, Information and Autonomy. *Theor Med Bioeth* 2001; 22: 415–436. L. Bortolotti. The Relative Importance of Undesirable Truths. *Med Health Care Philos* 2013; 16: 683–690. 10.1007/s11019-012-9449-xPMC383275823160857

28
In the study family those samples were requested from all family members and led to the ‘discovery’ of Lynch Syndrome.

29

K. Finkler , et al. The New Genetics and its Consequences for Family, Kinship, Medicine and Medical Genetics. Soc Sci Med 2003; 57: 403–412.410. 1279148410.1016/s0277-9536(02)00365-9

30
Widdows, Harris & Keywood, Bortolotti, *op. cit*. note 27.

31

L. Bortolotti , P. Malpas . The Right to Remain in Ignorance about Genetic Information ‐ Can Such a Right be Defended in the Name of Autonomy? N Z Med J 2005; 118: U1611–U1611. 16132072

32
Bortolotti, *op. cit* note 27; J. Harris & K. Keywood. Ignorance, Information and Autonomy. *Theor Med Bioeth* 2001; 22: 415–436. 10.1023/a:101305880162211808677

33
Bortolotti, *op. cit*. note 27, p.689.

34
A. Erez, et al. The Right to Ignore Genetic Status of Late Onset Genetic Disease in the Genomic Era; Prenatal Testing for Huntington Disease as a Paradigm. *Am J Med Genet* 2010; Part A: 1774–1780. 10.1002/ajmg.a.33432PMC364884220583190

35
C. Dancyger, et al. Comparing Family Members' Motivations and Attitudes Towards Genetic Testing for Hereditary Breast and Ovarian Cancer: A Qualitative Analysis. *Eur. J. Hum. Genet* 2010; 18: 1289–1295; D. Sacchini, et al. 2002. *Genetic Screening ‐ Benefits and Pitfalls*. Bratislava: Charis: 131–151. 10.1038/ejhg.2010.114PMC300286220648056

36

Plummer K. 2001 The Call of Life Stories in Ethnographic Research In Handbook of Ethnography. AtkinsonP., CoffeyA., DelamontS., LoflandJ. and LoflandL, eds. London: Sage: 395–406. Singer

